# Supplementary material for: Blue Turns to Gray: Paleogenomic Insights into the Evolutionary History and Extinction of the Blue Antelope (Hippotragus leucophaeus)
Source: Mol Biol Evol. 2022 Nov 2;39(12):msac241. doi: 10.1093/molbev/msac241 (PMC9750129; doi:10.1093/molbev/msac241)
Supplement: msac241_Supplementary_Data [file msac241_supplementary_data.zip › S1_Supplementary_File_S1.pdf]

Blue turns to gray – Paleogenomic insights into the evolutionary history and extinction of the blue antelope (*Hippotragus leucophaeus*)

Elisabeth Hempel, Faysal Bibi, J. Tyler Faith, Klaus-Peter Koepfli, Achim M. Klittich, David A. Duchêne, James S. Brink, Daniela C. Kalthoff, Love Dalén, Michael Hofreiter and Michael V. Westbury

Supplementary figures

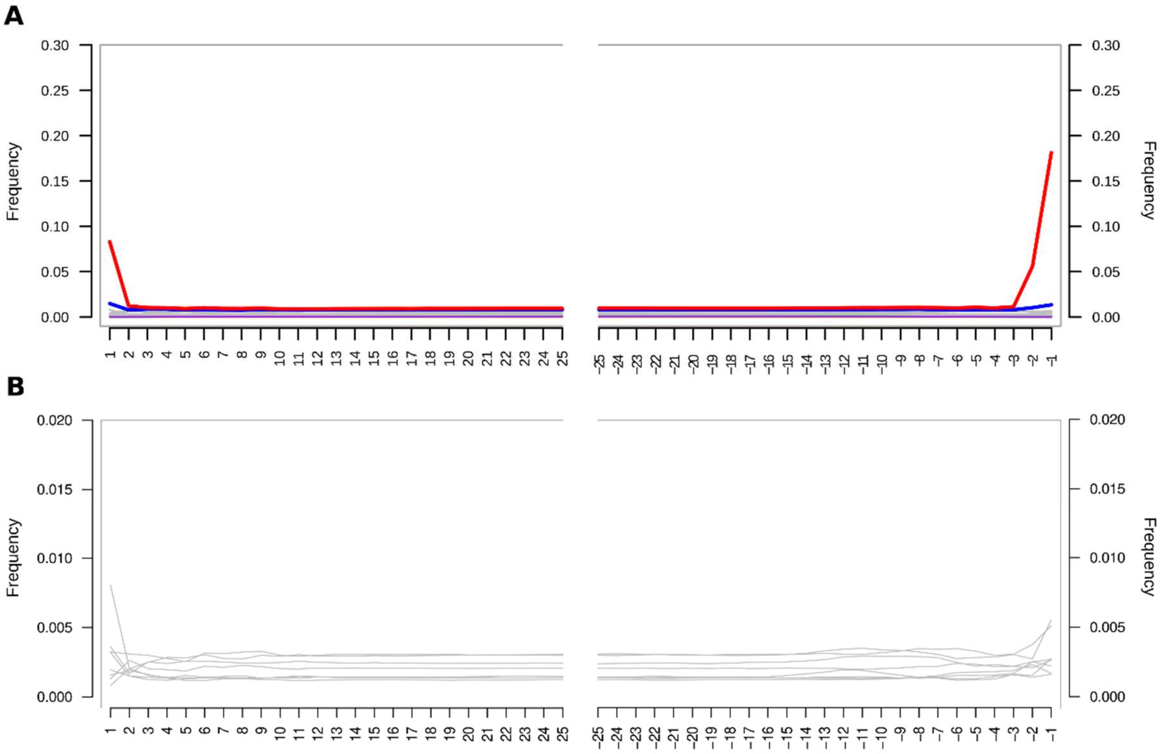

Supplementary fig. S1. DNA damage patterns for blue antelope specimen NBC RB4 D3 using mapDamage v2.2.0 (Jónsson et al. 2013). (**A**) MapDamage output, red: cytosine to thymine substitutions, blue: guanine to adenine substitutions, gray: all other substitutions. As expected from sequences from single-stranded library preparation, no guanine to adenine substitutions are shown on the 3’ end (right) (Meyer et al. 2012) (**B**) Modified mapDamage output displaying only transversions. No transversion is distinctly higher. Note the different scales on the y-axes in A and B.

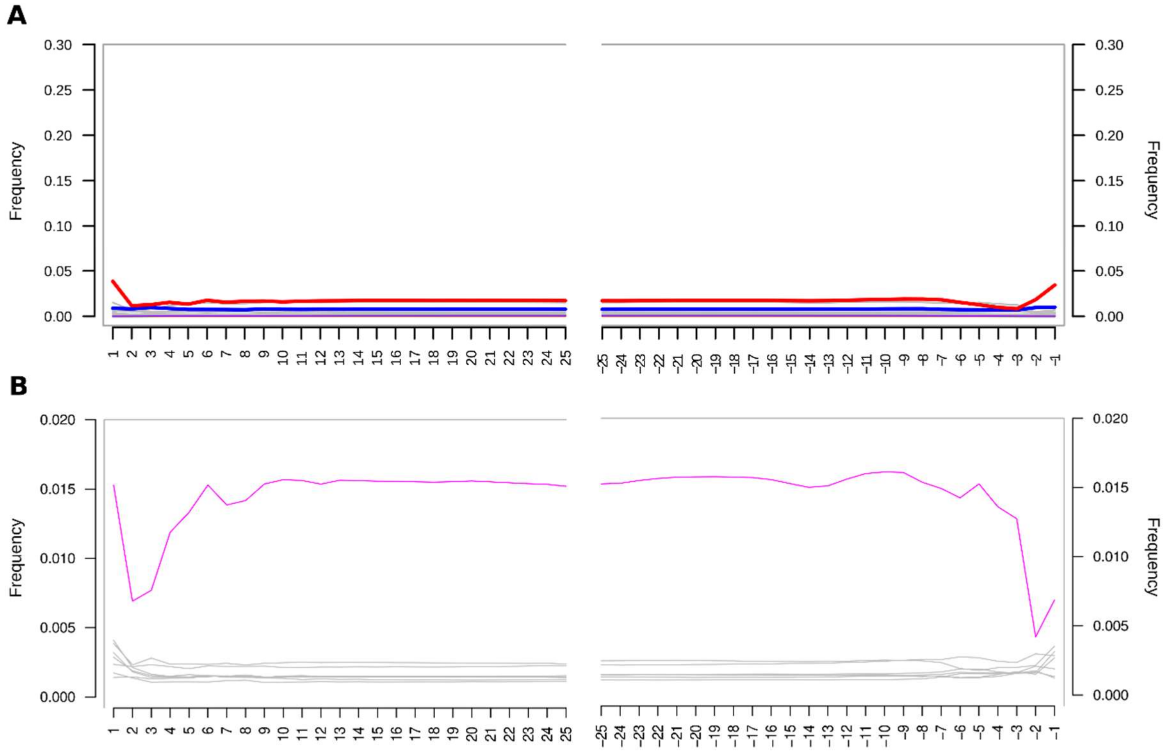

Supplementary fig. S2. DNA damage patterns for blue antelope specimen NRM 590107 using mapDamage v2.2.0 (Jónsson et al. 2013). (**A**) MapDamage output, red: cytosine to thymine substitutions, blue: guanine to adenine substitutions, gray: all other substitutions. As expected from sequences from single-stranded library preparation no guanine to adenine substitutions are shown on the 3' end (right) (Meyer et al. 2012) (**B**) Modified mapDamage output displaying only transversions, showing elevated guanine to thymine levels (pink). Patterns like this could originate from hydrogen peroxide treatment of the sample (Kvam and Tyrrell 1997; Nohmi et al. 2005). Note the different scales on the y-axes in A and B.

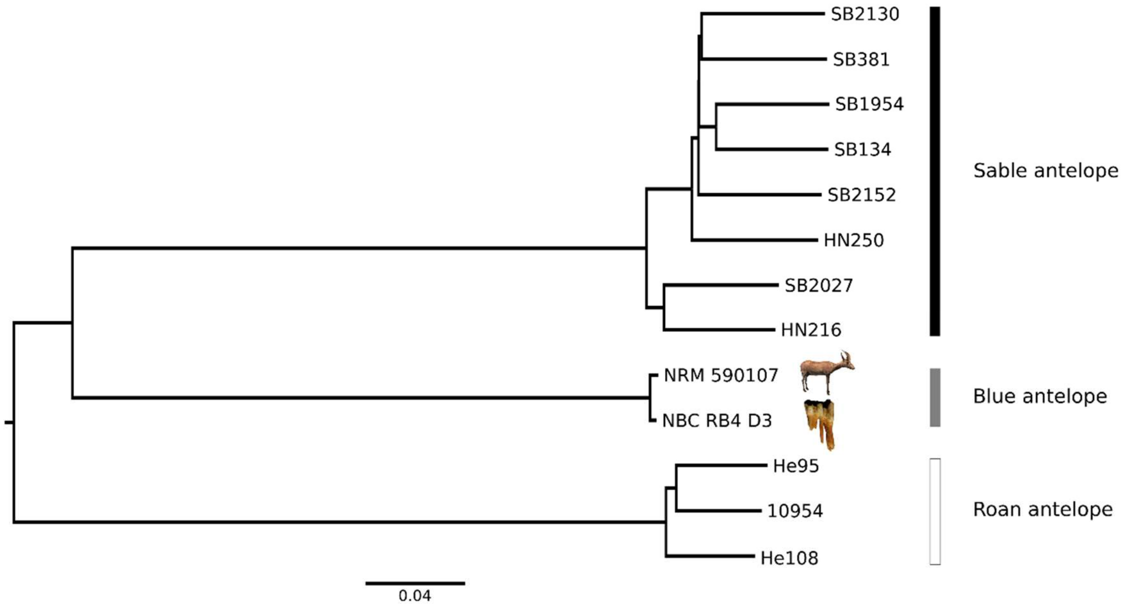

Supplementary fig. S3. Neighbor joining phylogenetic tree (singletons removed) of the three *Hippotragus* species based on nuclear genomes showing the sister relationship of blue and sable antelope. The phylogeny was constructed using ANGSD v0.923 (Korneliussen et al. 2014) and FastME v2.1.6.1 (Lefort et al. 2015). The scimitar-horned oryx (SB20612, Humble et al. 2020) was used as outgroup (not shown). Roan and sable antelope raw data are from Gonçalves et al. (2021) and Koepfli et al. (2019). Photo credits: NBC RB4 D3: J.T. Faith, courtesy: Archaeology Unit, Iziko Museums of South Africa; NRM 590107: Hempel, Bibi, et al. 2021.

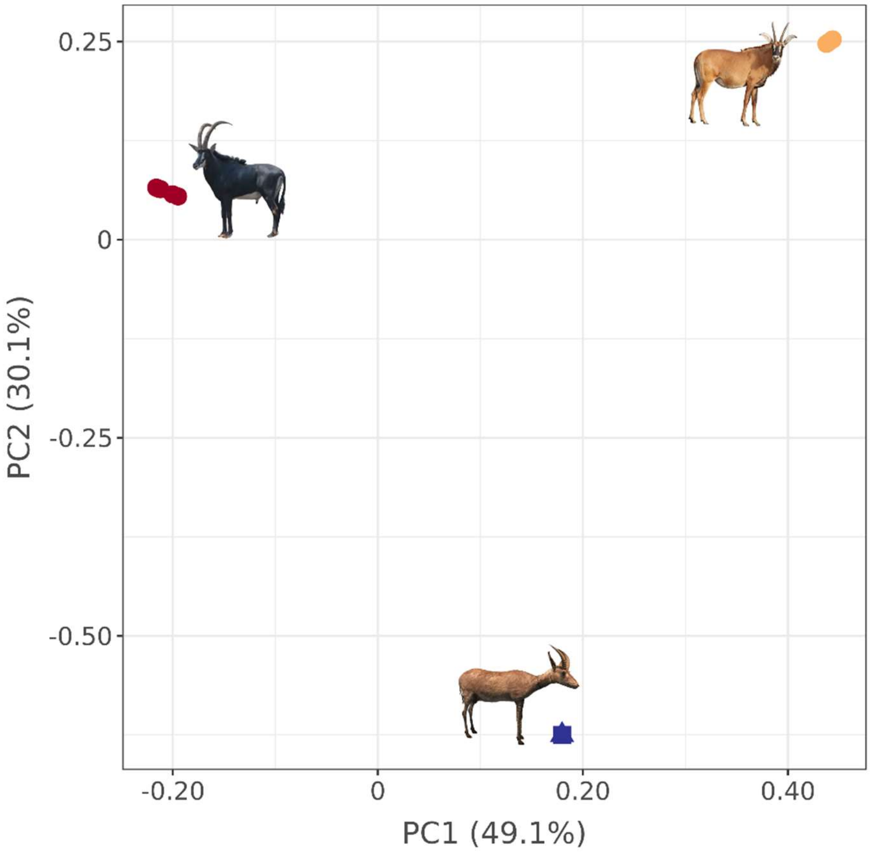

Supplementary fig. S4. Principal component analysis displaying the clustering of two blue (indicated in dark blue), three roan (indicated in orange) and eight sable antelope samples (indicated in red). Circles represent contemporary samples, the square represents the historical and the triangle the early Holocene sample. The analysis was conducted in ANGSD v0.923 (Korneliussen et al. 2014). Photo credits: NRM 590107: Hempel, Bibi, et al. 2021, roan antelope: Charles J. Sharp, wikimedia commons, CC-BY 4.0; sable antelope: Paulmaz, wikimedia commons, CC-BY 3.0.

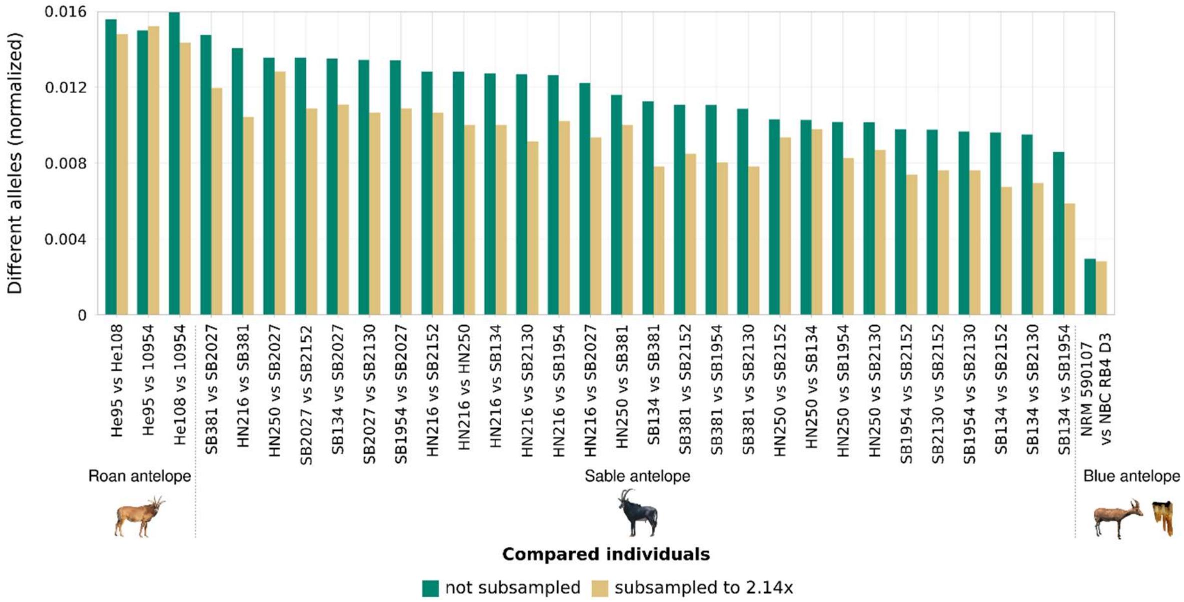

Supplementary fig. S5. Species-wide nuclear genome diversity of the three *Hippotragus* species computed from pairwise comparisons for data subsampled to 2.14x mean coverage (yellow bars) and data not subsampled (green bars). Data normalized for 4,596 and 2,243,953 sites, respectively. Genome data of roan and sable antelope are from Gonçalves et al. (2021) and Koepfli et al. (2019), respectively. Photo credits: NBC RB4 D3: J.T. Faith, courtesy: Archaeology Unit, Iziko Museums of South Africa; NRM 590107: Hempel, Bibi, et al. 2021, roan antelope: Charles J. Sharp, wikimedia commons, CC-BY 4.0; sable antelope: Paulmaz, wikimedia commons, CC-BY 3.0.

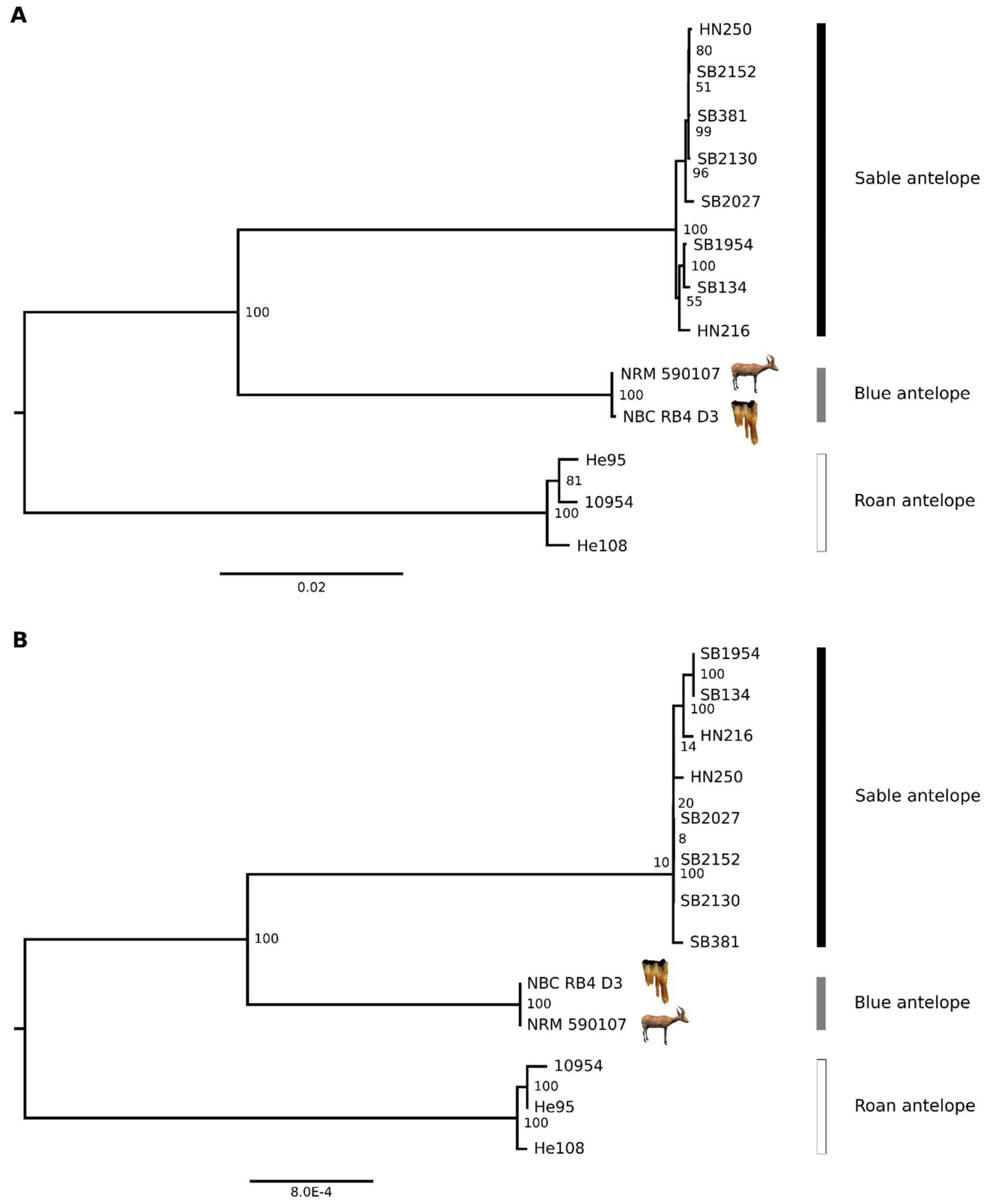

Supplementary fig. S6. Mitochondrial maximum-likelihood phylogeny. (**A**) Maximum-likelihood phylogeny of mitochondrial genomes of the three *Hippotragus* species. The phylogeny was generated using RAxML v8.2.12 with a GTR + G substitution model (Stamatakis 2014). The control region was excluded from the alignment (15,440 bp alignment length). (**B**) Maximum-likelihood phylogeny of mitochondrial genomes of the three *Hippotragus* species. The phylogeny is based on transversions only and was generated using RAxML with a BINGAMMA substitution model. The control region was excluded from the alignment (15,440 bp alignment length, non-binary format). Node labels show bootstrap support from 1,000 replicates. In both analyses, the scimitar-horned oryx (*Oryx dammah*, SB20612, Humble et al. 2020) was used as outgroup (not shown). Mitochondrial genome sequences of roan and sable antelope generated from Gonçalves et al. (2021) and Koepfli et al. (2019). Photo credits: see supplementary fig. S3.

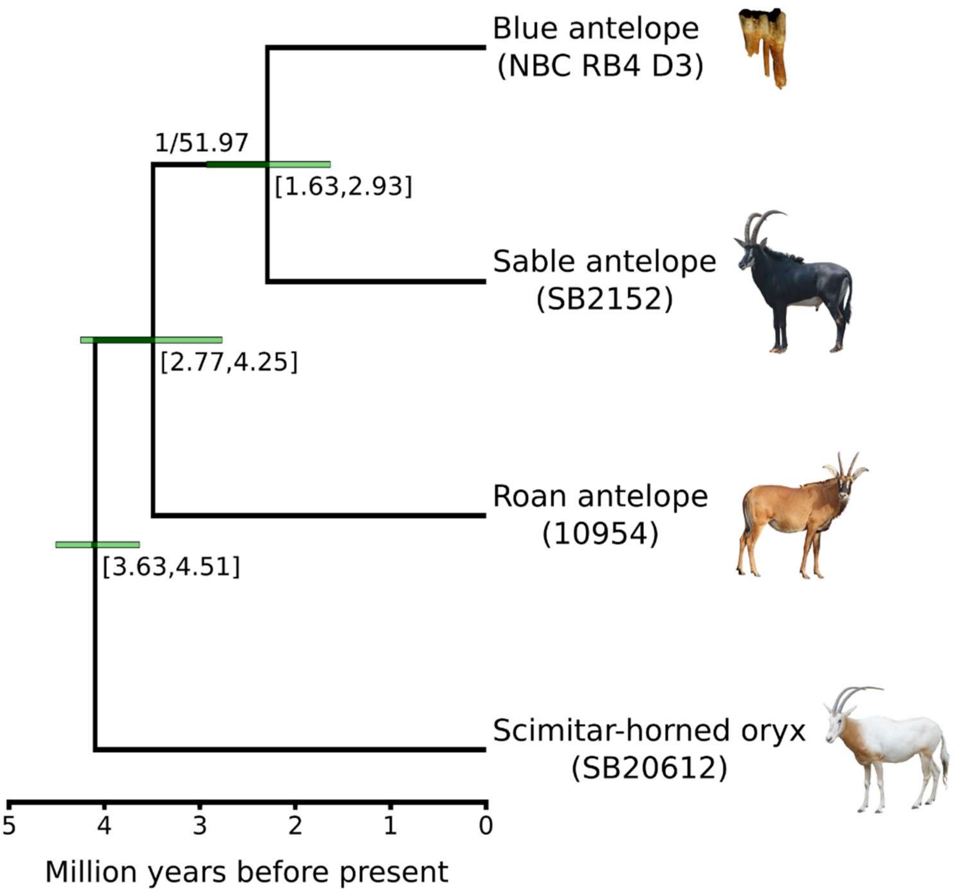

Supplementary fig. S7. Mitochondrial fossil-calibrated Bayesian phylogeny. Dated phylogenetic tree computed using mitochondrial data from the three *Hippotragus* species. The tree topology was inferred using a data set that excluded transition-only sites, under the multi-species coalescent model using ASTRAL v4.10 (Rabiee et al. 2019), while the timescale was inferred using MCMCtree v4.9 (dos Reis and Yang 2011). Node annotations show the respective credibility interval's range in Mya, while the branch label shows support values (bootstrap, site concordance factor). Branch support is calculated on an unrooted tree, in this case with four taxa, so the support is for the separation between two pairs of taxa as represented by that single internal branch. The control region was excluded from the alignment (15,440 bp alignment length, non-binary format). The scimitar-horned oryx was used as outgroup (Humble et al. 2020). Mitochondrial genome sequences of roan and sable antelope generated from Gonçalves et al. (2021) and Koepfli et al. (2019). Photo credits: NBC RB4 D3: J.T. Faith, courtesy: Archaeology Unit, Iziko Museums of South Africa; roan antelope: Charles J. Sharp, wikimedia commons, CC-BY 4.0; sable antelope: Paulmaz, wikimedia commons, CC-BY 3.0; scimitar-horned oryx: E. Hempel.

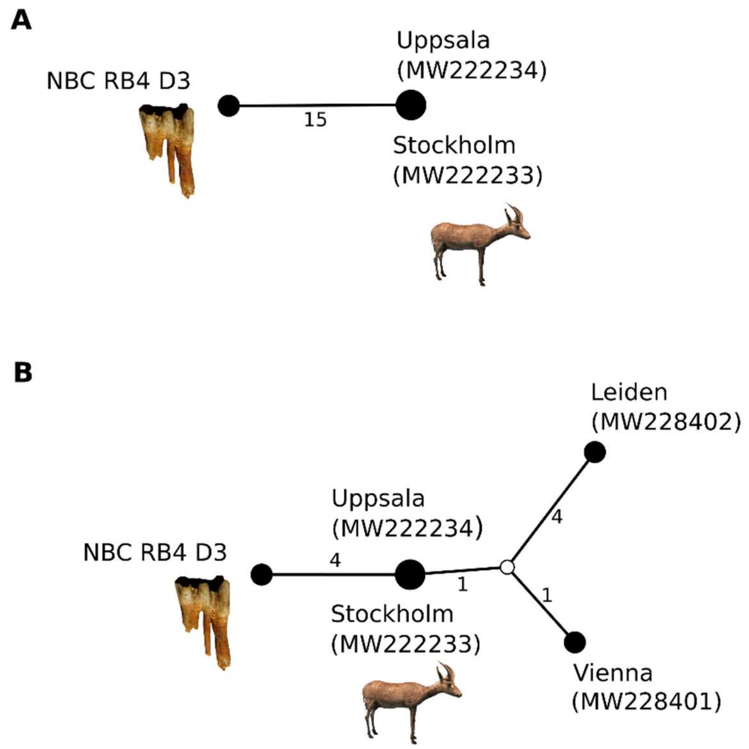

Supplementary fig. S8. Blue antelope mitochondrial haplotype networks. (**A**) Median-joining network of three complete blue antelope mitochondrial genomes (alignment length 16,492 bp) generated in POPART v1.7 (Bandelt et al. 1999; Leigh and Bryant 2015). Ambiguities/missing data were excluded. Numbers at lines represent mutational steps. Circle sizes correspond to frequency of haplotypes. City names are locations of the museum specimens, GenBank accession numbers are in parentheses. (**B**) Median-joining network of three complete and two partial mitochondrial genomes (alignment length 6,300 bp). The white circle represents an unsampled haplotype. Photo credits: see supplementary fig. S3.

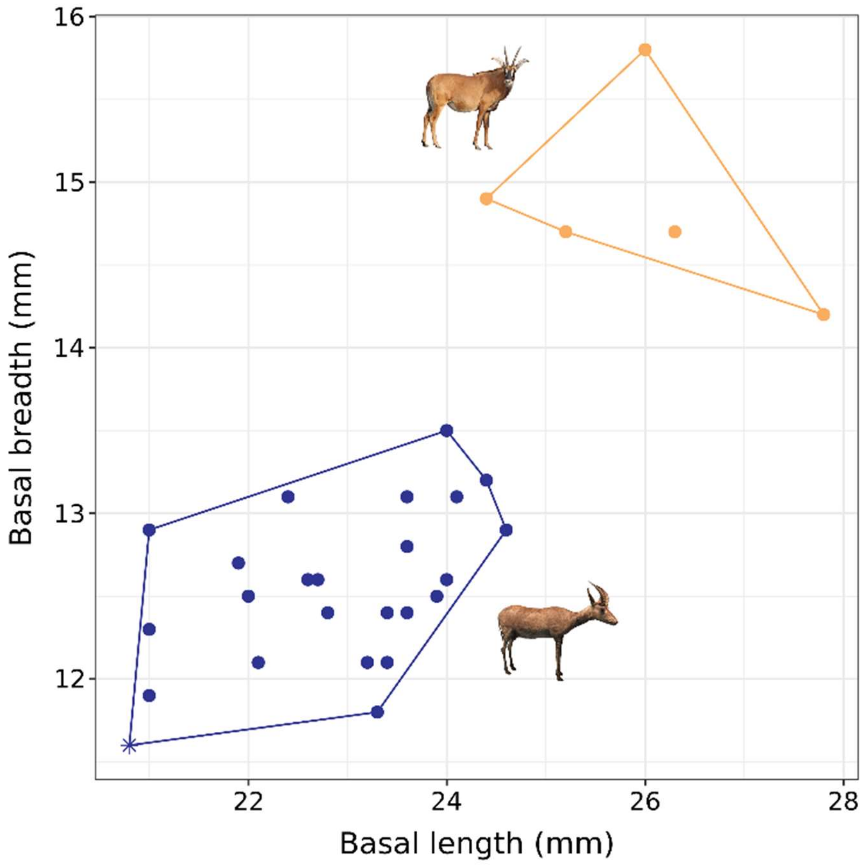

Supplementary fig. S9. Bivariate plot of basal length versus basal width of blue (indicated in dark blue) and roan antelope (indicated in orange) lower fourth deciduous premolars from late Quaternary archaeological sites in the Western Cape, South Africa. Roan antelope specimens are from Boomplaas Cave and Nelson Bay Cave; blue antelope specimens are from Boomplaas Cave, Blombos Cave, Die Kelders Cave 1, Nelson Bay Cave, and Klasies River Mouth 1 (see supplementary table S18). Length and width measurements (in mm) taken at the base of the tooth crown. Nelson Bay Cave specimen NBC RB4 D3 is indicated with an asterisk. Photo credits: see supplementary fig. S4.

Supplementary tables

Supplementary table S1. Sample information for the fossil blue antelope (*Hippotragus leucophaeus*) specimens housed at the Archaeology Unit, Iziko Museums of South Africa, Cape Town, South Africa, from which DNA was extracted in this study. The sample from which DNA was extracted successfully is printed in blue.

Site	Sample stratification number	Species identification	Age of layer [cal years BP]	Sampled material	References
Elands Bay Cave	EBC KM E6	*Hippotragus leucophaeus*	14,000–12,000	Tooth root (P^4)	Klein and Cruz-Uribe 2016; Parkington 2016
Die Kelders Cave 1	DK1 M/J D3	*Hippotragus* cf. *leucophaeus*	71,000–57,000	Tooth root (I_2)	Klein and Cruz-Uribe 2000; Marean et al. 2000
	DK1 MJA E5	*Hippotragus* cf. *leucophaeus*	71,000–57,000	Tooth root (I_1)	
Byneskranskop 1	BNK1 O29 7	*Hippotragus leucophaeus*	6,200–5,900	Mandible fragment	Schweitzer and Wilson 1982; Loftus et al. 2016
	BNK1 O29 5	*Hippotragus leucophaeus*	6,300–6,000	Tooth root (P^3)	
	BNK1 P29 19 QUI	*Hippotragus leucophaeus*	17,000–16,000	Tooth root (P_4)	
	BNK1 O25/2 5.2	*Hippotragus leucophaeus*	^{14}C 3881 ± 42[1]	For aDNA extraction: tooth root (M^2) & for carbon dating: maxilla	
Boomplaas Cave	BPA BGM CL3 016/61 4.4890	*Hippotragus leucophaeus*	17,800–13,700	Mandible	Klein 1978; Pargeter et al. 2018
	BPA BRL7 Q15/51 3.5127	*Hippotragus leucophaeus*	14,000–12,600	Mandible	
	BPA LPM P13 6.419	*Hippotragus leucophaeus*	23,500–21,400	Mandible	

Nelson Bay Cave	NBC BSBI 9 D9	*Hippotragus leucophaeus*	7,000–6,200	Mandible	Klein 1983; Loftus et al. 2016
	NBC RA 9 B1	*Hippotragus leucophaeus*	9,200–6,900	Mandible	
	NBC RB 4 D3	*Hippotragus leucophaeus*	9,800–9,300	Tooth root (dP_4)	
	NBC J 1 D5	*Hippotragus leucophaeus*	11,400–9,500	Tooth root (M^3)	
	NBC BSJ 80 B4	*Hippotragus leucophaeus*	11,900–10,900	Mandible	
	NBC CS 2 D5	*Hippotragus leucophaeus*	12,200–11,700	Mandible	
	NBC CS 52 D8	*Hippotragus leucophaeus*	12,200–11,700	Mandible	
	NBC BSTL 26 D3	*Hippotragus leucophaeus*	14,900–12,000	Tooth root (M^1)	
	NBC GBSL 43	*Hippotragus leucophaeus*	14,900–12,000	Tooth root (P^2)	
	NBC YSL 8 G8	*Hippotragus leucophaeus*	22,100–16,300	Tooth root (M_1)	
	NBC YGL 20 D5	*Hippotragus leucophaeus*	23,500–21,000	Maxilla	
	NBC DS 18 1	*Hippotragus leucophaeus*	Stratigraphic position unclear	Mandible	
Klasies River Mouth 1/1A	KRM1 13 ??? 2071	*Hippotragus leucophaeus*	~65,000–60,000	Tooth root (P^4)	Klein 1976; Singer and Wymer 1982; Grine et al. 2017
	KRM1A 9C 41459	*Hippotragus leucophaeus*	~60,000	Mandible	
	KRM1A 6 1A 40.42	*Hippotragus leucophaeus*	~60,000	Tooth root (M_2)	

[1] Radiocarbon dated in this study calibrated using Reimer et al. (2013) and CALIB REV 7.0.0 (Stuiver and Reimer 1993)

Supplementary table S2. Paired-end (PE) and single-end (SE) sequencing results from the fossil (NBC RB4 D3) and historical (NRM 590107) blue antelope (*Hippotragus leucophaeus*) samples mapped to the nuclear genome assembly of the scimitar-horned oryx (*Oryx dammah*, https://www.dnazoo.org/assemblies/Oryx_dammah, Humble et al. 2020). Only merged reads were used. The paired-end run of NBC RB4 D3 was split by the Sequence Read Archive into four files.

see supplementary file S2

Supplementary table S3. Paired-end (PE) and single-end (SE) sequencing results from the fossil (NBC RB4 D3) blue antelope sample mapped to the mitochondrial genome of the blue antelope (*Hippotragus leucophaeus*, MW222233, Hempel, Bibi, et al. 2021). Only merged reads were used. “Shifted” refers to the second mapping to a reference with 200 bp shifted from the end to the beginning of the reference. The paired-end run of NBC RB4 D3 was split by the Sequence Read Archive into four files.

see supplementary file S2

Supplementary table S4. Paired-end sequencing results for the roan (*Hippotragus equinus*, Gonçalves et al. 2021) and sable antelope (*Hippotragus niger*, Koepfli et al. 2019) and the scimitar-horned oryx (*Oryx dammah*, Humble et al. 2020), mapped to the nuclear genome assembly of the scimitar-horned oryx (https://www.dnazoo.org/assemblies/Oryx_dammah, Humble et al. 2020). Merged and unmerged reads were used.

see supplementary file S2

Supplementary table S5. Paired-end sequencing results for the roan (*Hippotragus equinus*) (Gonçalves et al. 2021) and sable antelope (*Hippotragus niger*) (Koepfli et al. 2019) and the scimitar-horned oryx (*Oryx dammah*) (Humble et al. 2020) mapped to the mitochondrial genome assembly of the roan and sable antelope and the scimitar-horned oryx (JN632647, JN632648, JN632677; Hassanin et al. 2012). Merged and unmerged reads were used. "Shifted" refers to the second mapping to a reference with base pairs shifted from the end to the beginning of the reference (290bp for the scimitar-horned oryx, 252bp for the sable antelope and 300bp for the roan antelope reference).

see supplementary file S2

Supplementary table S6. Autosomal scaffold IDs >1 Mb with scaffold size in parentheses of the scimitar-horned oryx (*Oryx dammah*) used in this study. Autosomal scaffolds were used as determined in Hempel, Westbury, et al. (2021) by aligning the genome of the scimitar-horned oryx (Humble et al. 2020) to the X chromosome of the domestic goat (CM001739.2, Dong et al. 2013), the Y chromosome of the wild goat (CM003213.1, Dong et al. 2015) and the mitochondrial genome of the scimitar-horned oryx (JN632677, Hassanin et al. 2012) using SatsumaSynteny v2.0 (Grabherr et al. 2010).

**Scaffold IDs >1 Mb of scimitar-horned oryx**	
HiC_scaffold_1 (111,031,402 bp),<br>HiC_scaffold_4 (90,666,804 bp),<br>HiC_scaffold_5 (107,200,824 bp),<br>HiC_scaffold_8 (61,296,289 bp),<br>HiC_scaffold_9 (60,415,816 bp),<br>HiC_scaffold_10 (82,229,679 bp),<br>HiC_scaffold_11 (51,053,030 bp),<br>HiC_scaffold_13 (64,050,454 bp),<br>HiC_scaffold_14 (50,506,852 bp),<br>HiC_scaffold_16 (67,964,418 bp),<br>HiC_scaffold_20 (71,114,058 bp),<br>HiC_scaffold_25 (105,560,413 bp),<br>HiC_scaffold_26 (100,398,400 bp),<br>HiC_scaffold_28 (118,425,367 bp),<br>HiC_scaffold_11045 (1,665,326 bp),	HiC_scaffold_11050 (12,440,347 bp),<br>HiC_scaffold_11056 (3,214,039 bp),<br>HiC_scaffold_11058 (2,359,909 bp),<br>HiC_scaffold_11072 (3,672,348 bp),<br>HiC_scaffold_11077 (8,331,692 bp),<br>HiC_scaffold_11081 (3,557,701 bp),<br>HiC_scaffold_11151 (1,109,432 bp),<br>HiC_scaffold_11163 (6,504,183 bp),<br>HiC_scaffold_11164 (7,448,190 bp),<br>HiC_scaffold_11203 (7,326,413 bp),<br>HiC_scaffold_11209 (5,441,144 bp),<br>HiC_scaffold_11210 (8,020,263 bp),<br>HiC_scaffold_11227 (4,755,615 bp),<br>HiC_scaffold_11229 (6,595,699 bp),<br>HiC_scaffold_11232 (7,227,722 bp)

Supplementary table S7. *D* values, Z-scores, number of ABBA and BABA sites, and weighted block jacknife *D* values from computing *D* statistics using Dstat (transversion only version, https://github.com/jacahill/Admixture) with individuals in the following arrangement: P1: sable antelope (SB2152, Koepfli et al. 2019), P2: blue antelope, P3: roan antelope (10954, Gonçalves et al. 2021), P4: scimitar-horned oryx (SB20612, Humble et al. 2020).

**Blue antelope specimen**	***D* value**	**# ABBA sites**	**# BABA sites**	**Weighted block jackknife D value**	**Z-score**
NBC RB4 D3	0.1444	66,054	49,380	0.0073	19.7664
NRM 590107	0.1240	75,996	59,227	0.0070	17.6837

Supplementary table S8. Number of trees found for sliding window tree analyses using WindowTrees v1.0.0 (https://github.com/achimklittich/WindowTrees) to determine gene flow with a window size of 100 kb and a threshold for missing data of 50% in binary mode with the individuals of blue antelope (NBC RB4 D3, NRM 590107, both this study), sable antelope (SB2152, HN216; Koepfli et al. 2019) and roan antelope (10954, He108; Gonçalves et al. 2021). The scimitar-horned oryx was used as outgroup (SB20612, Humble et al. 2020).

**10954, He108, SB2152, NBC RB4 D3**	
(((SB2152,NBCRB4D3),(10954,He108)),SB20612)	7,003
(((NBCRB4D3,(10954,He108)),SB2152),SB20612)	2,705
((NBCRB4D3,((10954,He108),SB2152)),SB20612)	1,115
(((He108,(SB2152,NBCRB4D3)),10954),SB20612)	12
(((SB2152,(He108,NBCRB4D3)),10954),SB20612)	4
sum	10,839
**10954, He108, SB2152, NRM 590107**	
(((SB2152,NRM590107),(10954,He108)),SB20612)	6,802
(((NRM590107,(10954,He108)),SB2152),SB20612)	2,923
((NRM590107,((10954,He108),SB2152)),SB20612)	1,068
(((He108,(SB2152,NRM590107)),10954),SB20612)	12
(((SB2152,(He108,NRM590107)),10954),SB20612)	4
((((NRM590107,He108),10954),SB2152),SB20612)	1
Sum	10,810
**10954, He108, HN216, NRM590107**	
(((HN216,NRM590107),(10954,He108)),SB20612)	6,819
(((NRM590107,(10954,He108)),HN216),SB20612)	2,936
((NRM590107,((10954,He108),HN216)),SB20612)	1,038
(((He108,(HN216S,NRM590107)),10954),SB20612)	11
(((HN216,(He108,NRM590107)),10954),SB20612)	4
(((10954,(He108,NRM590107)),HN216),SB20612)	1
sum	10,809

**10954, He108, HN216, NBCRB4D3**	
(((HN216,NBCRB4D3),(10954,He108)),SB20612)	6,976
(((NBCRB4D3,(10954,He108)),HN216),SB20612)	2,770
((NBCRB4D3,((10954,He108),HN216)),SB20612)	1,076
(((He108,(HN216,NBCRB4D3)),10954),SB20612)	10
(((HN216,(He108,NBCRB4D3)),10954),SB20612)	5
Sum	10,837

Supplementary table S9. Number of tree topologies found for the sliding window tree analysis to determine gene flow directionality using WindowTrees v1.0.0 (https://github.com/achimklittich/WindowTrees) with a window size of 20 kb, a gap size of 80 kb between windows and a missing data threshold of 50% in binary mode with one individual per species: roan antelope (10954, Gonçalves et al. 2021), blue antelope (NBC RB4 D3, this study), sable antelope (SB2152, Koepfli et al. 2019) and the scimitar-horned oryx (SB20612, Humble et al. 2020) as outgroup.

**Tree**	**Topology**	**# topologies found**
1	(((NBCRB4D3,10954),SB2152),SB20612)	3,209
2	(((NBCRB4D3,SB2152),10954),SB20612)	5,437
3	((NBCRB4D3,(SB2152,10954)),SB20612)	2,128

Supplementary table S10. Matrix of differing alleles for all pairwise comparisons for the eight sable antelope (*Hippotragus niger*) individuals using raw (below the diagonal)/normalized data (above the diagonal, data normalized by dividing by the number of sites used in the analysis – 2,243,953 sites) to estimate the species-wide nuclear diversity in relation to heterozygosity and to compare it to the other species. Normalized values plotted in fig. 5. Data used are from Koepfli et al. (2019).

	**HN216**	**HN250**	**SB134**	**SB381**	**SB1954**	**SB2027**	**SB2130**	**SB2152**
**HN216**	x	0.01283	0.01272	0.01406	0.01265	0.01222	0.0127	0.01283
**HN250**	28,784	x	0.01028	0.01160	0.01018	0.01355	0.01016	0.01032
**SB134**	28,539	23,060	x	0.01126	0.00858	0.01352	0.00950	0.00962
**SB381**	31,545	26,035	25,277	x	0.01106	0.01477	0.01087	0.01107
**SB1954**	28,396	22,838	19,262	24,822	x	0.01342	0.00967	0.00977
**SB2027**	27,428	30,409	30,344	33,132	30,118	x	0.01344	0.01355
**SB2130**	28,491	22,801	21,323	24,388	21,702	30,167	x	0.00977
**SB2152**	28,792	23,151	21,594	24,839	21,931	30,407	21,917	x

Supplementary table S11. Matrix of differing alleles for all pairwise comparisons for the three roan antelope (*Hippotragus equinus*) individuals using raw (below the diagonal)/normalized data (above the diagonal, data normalized by dividing by the number of sites used in the analysis – 2,243,953 sites) to estimate the species-wide nuclear diversity in relation to heterozygosity and to compare it to the other species. Normalized values plotted in fig. 5. Data used are from Gonçalves et al. (2021).

	**He95**	**He108**	**10954**
**He95**	x	0.01558	0.01500
**He108**	34,958	x	0.01595
**10954**	33,664	35,791	x

Supplementary table S12. Matrix of differing alleles for all pairwise comparisons for the two blue antelope (*Hippotragus leucophaeus*) individuals using raw (below the diagonal)/normalized data (above the diagonal, data normalized by dividing by the number of sites used in the analysis – 2,243,953 sites) to estimate species-wide nuclear diversity and to compare it to the other species. Normalized value plotted in fig. 5.

	NRM 590107	NBC RB4 D3
**NRM 590107**	x	0.00296
**NBC RB4 D3**	6,649	x

Supplementary table S13. Matrix of differing alleles for all pairwise comparisons for the eight sable antelope (*Hippotragus niger*) individuals using raw (below the diagonal)/normalized data (above the diagonal, data normalized by dividing by the number of sites used in the analysis – 4,596 sites) for genomes subsampled to 2.14x mean coverage to exclude the possible effect of sequence coverage on the species-wide nuclear diversity estimate. Data used are from Koepfli et al. (2019).

	HN216	HN250	SB134	SB381	SB1954	SB2027	SB2130	SB2152
**HN216**	x	0.01001	0.01001	0.01044	0.01023	0.00936	0.00914	0.01066
**HN250**	46	x	0.00979	0.01001	0.00827	0.01284	0.00870	0.00936
**SB134**	46	45	x	0.00783	0.00587	0.0111	0.00696	0.00675
**SB381**	48	46	36	x	0.00805	0.01197	0.00783	0.00849
**SB1954**	47	38	27	37	x	0.01088	0.00762	0.0074
**SB2027**	43	59	51	55	50	x	0.01066	0.01088
**SB2130**	42	40	32	36	35	49	x	0.00762
**SB2152**	49	43	31	39	34	50	35	x

Supplementary table S14. Matrix of differing alleles for all pairwise comparisons for the three roan antelope (*Hippotragus equinus*) individuals using raw (below the diagonal)/normalized data (above the diagonal, data normalized by dividing by the number of sites used in the analysis – 4,596 sites) for genomes subsampled to 2.14x mean coverage to exclude the possible effect of sequence coverage on the species-wide nuclear diversity estimate. Data used are from Gonçalves et al. (2021).

	He95	He108	10954
**He95**	x	0.0148	0.01523
**He108**	68	x	0.01436
**10954**	70	66	x

Supplementary table S15. Matrix of differing alleles for all pairwise comparisons for the two blue antelope (*Hippotragus leucophaeus*) individuals using raw (below the diagonal)/normalized data (above the diagonal, data normalized by dividing by the number of sites used in the analysis – 4,596 sites) for genomes subsampled to 2.14x mean coverage to exclude the possible effect of sequence coverage for the species-wide nuclear diversity estimate.

	NRM 590107	NBC RB4 D3
**NRM 590107**	x	0.00283
**NBC RB4 D3**	13	x

Supplementary table S16. Estimated autosomal heterozygosity for all scaffolds >1 Mb computed in ANGSD v0.923 (Korneliussen et al. 2014) for all roan and sable antelope individuals using 1) non-subsampled data, or 2) data subsampled once to that of the individual of each species with the lowest mean coverage (roan: 7.18x, sable antelope: 4.26x), and 3) data subsampled once to the level of the individual with the lowest mean coverage in the roan/sable antelope data set (4.26x). Data for roan and sable antelope from Gonçalves et al. (2021) and Koepfli et al. (2019), respectively.

Sample ID	Species	Heterozygosity		
		**1) Not subsampled (mean coverage)**	**2) Subsampled to species level (mean coverage)**	**3) Subsampled to 4.26x**
10954	Roan antelope	0.00191 (24.42x)	0.00179 (7.18x)	0.00176
He95		0.00173 (7.18x)	0.00173 (7.18x)	0.00167
He108		0.00192 (7.40x)	0.00192 (7.18x)	0.00189
SB2152	Sable antelope	0.00152 (24.23x)	0.00129 (4.26x)	0.00129
SB134		0.00117 (4.58x)	0.00115 (4.26x)	0.00115
HN216		0.00171 (5.87x)	0.00167 (4.26x)	0.00167
HN250		0.00121 (6.78x)	0.00115 (4.26x)	0.00115
SB381		0.00154 (4.64x)	0.00152 (4.26x)	0.00152
SB1954		0.00121 (4.26x)	0.00121 (4.26x)	0.00121
SB2027		0.00111 (4.40x)	0.0011 (4.26x)	0.0011
SB2130		0.00124 (4.70x)	0.00121 (4.26x)	0.00121

Supplementary table S17. Mitochondrial sequences from GenBank included in the haplotype network built with POPART v1.7 (Bandelt et al. 1999; Leigh and Bryant 2015).

**Accession number**	**Sample ID**	**Included in network with … mitochondrial genomes**	**Publication**
ON101842	NBC RB4 D3	Complete & partial	this study
MW222233	NRM 590107	Complete & partial	Hempel, Bibi, et al. 2021
MW222234	UPS ZMC 78488	Complete & partial	Hempel, Bibi, et al. 2021
MW228401	NMW ST 715	Partial	Hempel, bibi, et al. 2021
MW228402	RMNH.MAM.20681.a	Partial	Hempel, Bibi, et al. 2021

Supplementary table S18. Sample information for the measured fossil blue (*Hippotragus leucophaeus*) and roan antelope (*Hippotragus equinus*) lower fourth deciduous premolars housed at the Archaeology Unit, Iziko Museums of South Africa, Cape Town, South Africa. The sample from which DNA was extracted successfully is printed in blue.

Site	Specimen number	Species identification	*Side*
Die Kelders Cave 1	DK1 MJA/H6	*Hippotragus leucophaeus*	Right
	DK1 6084	*Hippotragus leucophaeus*	Left
	DK1 -	*Hippotragus leucophaeus*	Right
Blombos Cave	BBC G5b 99.3	*Hippotragus leucophaeus*	Left
Boomplaas Cave	BPA O14/OCH3	*Hippotragus leucophaeus*	Left
Nelson Bay Cave	NBC YSL5 B8	*Hippotragus leucophaeus*	Left
	NBC YSL3 A3	*Hippotragus leucophaeus*	Right
	NBC DS19 1	*Hippotragus leucophaeus*	Left
	NBC RB4 D3	*Hippotragus leucophaeus*	Left
	NBC YGL34 A7	*Hippotragus leucophaeus*	Right
	NBC GBSL23 D6	*Hippotragus leucophaeus*	Left
Klasies River Mouth 1	KRM1 2050/1/14/ASP	*Hippotragus leucophaeus*	Right
	KRM1 2051/1/14/A	*Hippotragus leucophaeus*	Left
	KRM1 ?	*Hippotragus leucophaeus*	Left
	KRM1 20660	*Hippotragus leucophaeus*	Left
	KRM1 23856	*Hippotragus leucophaeus*	Left
	KRM1 2022	*Hippotragus leucophaeus*	Left
	KRM1 23855	*Hippotragus leucophaeus*	Right
	KRM1 200	*Hippotragus leucophaeus*	Right
	KRM1 201	*Hippotragus leucophaeus*	Left
	KRM1 23050	*Hippotragus leucophaeus*	Left
	KRM1 ?	*Hippotragus leucophaeus*	Right
	KRM1 2010	*Hippotragus leucophaeus*	Left
	KRM1 2011	*Hippotragus leucophaeus*	Left

Boomplaas Cave	BPA BRL6A P13	*Hippotragus equinus*	Right
Nelson Bay Cave	NBC BSJ4 B1	*Hippotragus equinus*	Right
	NBC CS16 D5	*Hippotragus equinus*	Right
	NBC CS23 D3	*Hippotragus equinus*	Right
	NBC BSJ105 B7	*Hippotragus equinus*	Right

References

Bandelt HJ, Forster P, Röhl A. 1999. Median-joining networks for inferring intraspecific phylogenies. *Mol Biol Evol*. **16**:37–48.

Dong Y, Xie M, Jiang Y, Xiao N, Du X, Zhang W, Tosser-Klopp G, Wang Jinhuan, Yang S, Liang J, et al. 2013. Sequencing and automated whole-genome optical mapping of the genome of a domestic goat (*Capra hircus*). *Nat Biotechnol*. **31**:135–141.

Dong Y, Zhang X, Xie M, Arefnezhad B, Wang Z, Wang Wenliang, Feng S, Huang G, Guan R, Shen W, et al. 2015. Reference genome of wild goat (*Capra aegagrus*) and sequencing of goat breeds provide insight into genic basis of goat domestication. *BMC Genomics*. **16**:431.

Gonçalves M, Siegismund HR, van Vuuren BJ, Koepfli K-P, Ferrand N, Godinho R. 2021. *De novo* whole-genome assembly and resequencing resources for the roan (*Hippotragus equinus*), an iconic African antelope. *G3* **11**:jkab002.

Grabherr MG, Russell P, Meyer M, Mauceli E, Alföldi J, Di Palma F, Lindblad-Toh K. 2010. Genome-wide synteny through highly sensitive sequence alignment: Satsuma. *Bioinformatics* **26**:1145–1151.

Grine FE, Wurz S, Marean CW. 2017. The Middle Stone Age human fossil record from Klasies River Main site. *J Hum Evol*. **103**:53–78.

Hassanin A, Delsuc F, Ropiquet A, Hammer C, Jansen van Vuuren B, Matthee C, Ruiz-Garcia M, Catzeflis F, Areskoug V, Nguyen TT, et al. 2012. Pattern and timing of diversification of Cetartiodactyla (Mammalia, Laurasiatheria), as revealed by a comprehensive analysis of mitochondrial genomes. *C R Biol*. **335**:32–50.

Hempel E, Bibi F, Faith JT, Brink JS, Kalthoff DC, Kamminga P, Paijmans JLA, Westbury MV, Hofreiter M, Zachos FE. 2021. Identifying the true number of specimens of the extinct blue antelope (*Hippotragus leucophaeus*). *Sci Rep*. **11**:2100.

Hempel E, Westbury MV, Grau JH, Trinks A, Paijmans JLA, Kliver S, Barlow A, Mayer F, Müller J, Chen L, et al. 2021. Diversity and paleodemography of the Addax (*Addax nasomaculatus*), a Saharan antelope on the verge of extinction. *Genes (Basel)*. **12**:1236.

Humble E, Dobrynin P, Senn H, Chuven J, Scott AF, Mohr DW, Dudchenko O, Omer AD, Colaric Z, Aiden EL, et al. 2020. Chromosomal-level genome assembly of the scimitar-horned oryx: Insights into diversity and demography of a species extinct in the wild. *Mol Ecol Resour*. **20**:1668–1681.

Jónsson H, Ginolhac A, Schubert M, Johnson PLF, Orlando L. 2013. mapDamage2.0: Fast approximate Bayesian estimates of ancient DNA damage parameters. *Bioinformatics* **29**:1682–1684.

Klein RG. 1976. The mammalian fauna of the Klasies River Mouth sites, southern Cape Province, South Africa. *S Afr Archaeol Bull*. **31**:75.

Klein RG. 1978. A preliminary report on the larger mammals from the Boomplaas Stone Age cave site, Cango Valley, Oudtshoorn district, South Africa. *S Afr Archaeol Bull*. **33**:66–75.

Klein RG. 1983. Palaeoenvironmental implications of Quaternary large mammals in the fynbos region. In: Deacon HJ, Hendrey QB, Lambrechts JNN, editors. *Fynbos paleoecology: A preliminary synthesis. South African National Scientific Programmes Rep*. Pretoria, Republic of South Africa: CSIR. p. 116–138.

Klein RG, Cruz-Uribe K. 2000. Middle and Later Stone Age large mammal and tortoise remains from Die Kelders Cave 1, Western Cape Province, South Africa. *J Hum Evol*. **38**:169–195.

Klein RG, Cruz-Uribe K. 2016. Large mammal and tortoise bones from Elands Bay Cave (South Africa): implications for Later Stone Age environment and ecology. *South Afr Humanit*. **29**:259–282.

Koepfli K-P, Tamazian G, Wildt D, Dobrynin P, Kim C, Frandsen PB, Godinho R, Yurchenko AA, Komissarov A, Krasheninnikova K, et al. 2019. Whole genome sequencing and re-sequencing of the sable antelope (*Hippotragus niger*): a resource for monitoring diversity in *ex situ* and *in situ* populations. *G3*. **9**:1785–1793.

Korneliussen TS, Albrechtsen A, Nielsen R. 2014. ANGSD: analysis of next generation sequencing data. *BMC Bioinform*. **15**:356.

Kvam E, Tyrrell RM. 1997. Artificial background and induced levels of oxidative base damage in DNA from human cells. *Carcinogenesis* **18**:2281–2283.

Lefort V, Desper R, Gascuel O. 2015. FastME 2.0: a comprehensive, accurate, and fast distance-based phylogeny inference program. *Mol Biol Evol*. **32**:2798–2800.

Leigh JW, Bryant D. 2015. POPART: full-feature software for haplotype network construction. *Methods Ecol Evol*. **6**:1110–1116.

Loftus E, Sealy J, Lee-Thorp J. 2016. New radiocarbon dates and Bayesian models for Nelson Bay Cave and Byneskranskop 1: implications for the South African Later Stone Age sequence. *Radiocarbon* **58**:365–381.

Marean CW, Goldberg P, Avery G, Grine FE, Klein RG. 2000. Middle Stone Age stratigraphy and excavations at Die Kelders Cave 1 (Western Cape Province, South Africa): the 1992, 1993, and 1995 field seasons. *J Hum Evol*. **38**:7–42.

Meyer M, Kircher M, Gansauge M-T, Li H, Racimo F, Mallick S, Schraiber J G, Jay F, Prüfer K, de Filippo C, et al. 2012. A high-coverage genome sequence from an archaic Denisovan individual. *Science* **338**:222–226.

Nohmi T, Kim S-R, Yamada M. 2005. Modulation of oxidative mutagenesis and carcinogenesis by polymorphic forms of human DNA repair enzymes. *Mutat Res*. **591**:60–73.

Pargeter J, Loftus E, Mackay A, Mitchell P, Stewart B. 2018. New ages from Boomplaas Cave, South Africa, provide increased resolution on late/terminal Pleistocene human behavioural variability. *Azania* **53**:156–184.

Parkington J. 2016. Elands Bay Cave: keeping an eye on the past. *South Afr Humanit*. **29**:17–32.

Rabiee M, Sayyari E, Mirarab S. 2019. Multi-allele species reconstruction using ASTRAL. *Mol Phylogenetics Evol*. **130**:186–296.

Reimer PJ, Bard E, Bayliss A, Beck JW, Blackwell PG, Ramsey CB, Buck CE, Cheng H, Edwards RL, Friedrich M, et al. 2013. IntCal13 and marine13 radiocarbon age calibration curves 0–50,000 years cal BP. *Radiocarbon* **55**:1869–1887.

dos Reis M, Yang Z. 2011. Approximate likelihood calculation on a phylogeny for Bayesian estimation of divergence times. *Mol Biol Evol*. **28**:2161–2172.

Schweitzer FR, Wilson ML. 1982. Byneskranskop 1. A late Quaternary living site in the southern Cape Province, South Africa. *Ann S Afr Mus*. **88**:1–203.

Singer R, Wymer J. 1982. *The Middle Stone Age at Klasies River Mouth in South Africa. Chicago*: Chicago University Press.

Stamatakis A. 2014. RAxML version 8: a tool for phylogenetic analysis and post-analysis of large phylogenies. *Bioinformatics* **30**:1312–1313.

Stuiver M, Reimer PJ. 1993. Extended ^{14}C data base and revised CALIB 3.0 ^{14}C age calibration program. *Radiocarbon* **35**:215–230.
