# Supplementary figures and images for: Blue Turns to Gray: Paleogenomic Insights into the Evolutionary History and Extinction of the Blue Antelope (Hippotragus leucophaeus)
Source: Mol Biol Evol. 2022 Nov 2;39(12):msac241. doi: 10.1093/molbev/msac241 (PMC9750129; doi:10.1093/molbev/msac241)

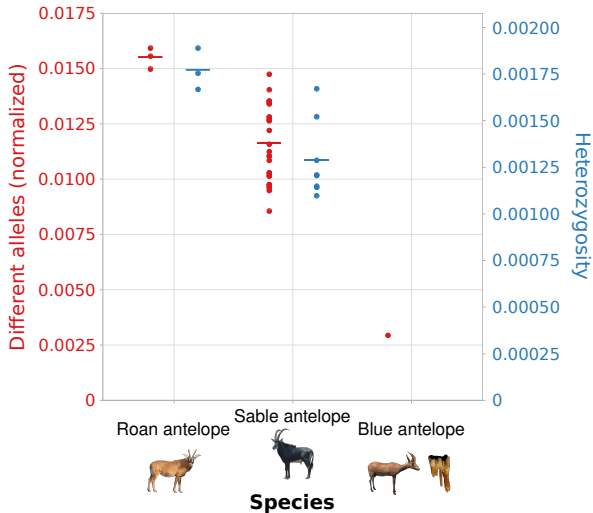

Supplement: msac241_Supplementary_Data [file msac241_supplementary_data.zip › Hempel_MBE-22-0326_Fig5_AE.pdf]
